# An Evaluability Assessment of the West Virginia Physical Activity Plan, 2015: Lessons Learned for Other State Physical Activity Plans

**DOI:** 10.5888/pcd13.160307

**Published:** 2016-12-29

**Authors:** Christiaan G. Abildso, Samantha Shawley, Sherry Owens, Angela Dyer, Sean M. Bulger, Dina L. Jones, Emily M. Jones, Emily Murphy, Melissa D. Olfert, Eloise Elliott

**Affiliations:** 1 West Virginia University School of Public Health, Morgantown, West Virginia; 2 West Virginia University College of Physical Activity and Sport Sciences, Morgantown, West Virginia; 3 West Virginia University Department of Orthopaedics and Division of Physical Therapy, Morgantown, West Virginia; 4 West Virginia University Extension Service, Morgantown, West Virginia; 5 West Virginia University Davis College of Agriculture, Natural Resources and Design, Department of Animal and Nutritional Science, Morgantown, West Virginia.

## Abstract

**Background:**

The US National Physical Activity Plan (NPAP) was released in 2009 as a national strategic plan to increase physical activity (PA). The NPAP emphasized implementing state and local PA programs. Dissemination of information about NPAP has been limited, however.

**Community Context:**

West Virginia is a predominantly rural state with high rates of chronic diseases associated with physical inactivity. In 2015 an evaluability assessment (EA) of the West Virginia Physical Activity Plan (WVPAP) was conducted, and community stakeholders were invited to participate in updating the plan.

**Methods:**

A good EA seeks stakeholder input, assists in identifying program areas that need improvement, and ensures that a full evaluation will produce useful information. Data for this EA were collected via national stakeholder interviews, document reviews, discussions among workgroups consisting of state and local stakeholders, and surveys to determine how well the WVPAP had been implemented.

**Outcome:**

The EA highlighted the need for WVPAP leaders to 1) establish a specific entity to implement local PA plans, 2) create sector-specific logic models to simplify the WVPAP for local stakeholders, 3) evaluate the PA plan’s implementation frequently from the outset, 4) use quick and efficient engagement techniques with stakeholders when working with them to select strategies, tactics, and measurable outcomes, and 5) understand the elements necessary to implement, manage, and evaluate a good PA plan.

**Interpretation:**

An EA process is recommended for other leaders of PA plans. Our project highlights the stakeholders’ desire to simplify the WVPAP so that it can be set up as a locally driven process that engages communities in implementation.

## Background

An estimated 10.8% of all-cause mortality in the United States can be attributed to insufficient physical activity (PA) (1). In 2008 the US Department of Health and Human Services published “Physical Activity Guidelines for Americans” (2), a national strategic plan that includes policies, practices, and initiatives that collectively could enable population increases in PA. A year later, *A National Physical Activity Plan for the United States* (NPAP) (3) was published, and in 2014 two articles were published (4,5) that emphasized implementing the NPAP as a state or local grassroots program. However, only Texas (6,7) and West Virginia (8,9) created stand-alone state PA plans; 9 other states included PA in their plans to decrease chronic disease or obesity. The lack of broad state and local dissemination of a plan to increase population PA suggests the existence of barriers already described in published articles (5,6,10,11); these barriers include the national plan’s complexity and lack of funding for implementation and evaluation.

## Community Context

West Virginia is a predominantly rural state of roughly 1.85 million people with high rates of poverty, residents aged 65 or older, and residents with lower rates of bachelor’s degrees than that of the United States as a whole (12); the population is mostly white. In addition, the prevalence of adults meeting PA guidelines in West Virginia is among the lowest in the United States, while the prevalence of chronic diseases associated with insufficient PA (including obesity, hypertension, type 2 diabetes, hypercholesterolemia, and cardiovascular disease) are among the highest (13,14).

Development of the West Virginia Physical Activity Plan (WVPAP) began in 2010, and it was intended to provide strategic direction for increasing PA opportunities and participation in the state. Modeled after the NPAP, the WVPAP was developed collaboratively, with a coordinating committee of experts leading teams that comprised stakeholders from 8 sectors: business and industry; education; health care; mass media; parks, recreation, fitness, and sports; public health; transportation, land use, and community design; and nonprofit or volunteer. A statewide event, the WV Physical Activity Symposium, was held in 2010 to explore the need for the plan, exchange ideas, and recruit stakeholders for sector teams. The event’s activities (including sector-specific concept mapping [15] exercises, a day-long work session for each sector team, and a public comment period) led to the launch of the WVPAP — *ActiveWV 2015* — on January 19, 2012 (9). 

The event also produced 5 cross-cutting priority areas to guide WVPAP implementation strategies: school programs and initiatives, public awareness and social marketing, community engagement and environment, institutional and organizational support, and policy. Each of the 8 sector teams developed 5 strategies — 1 for each priority area — and suggested tactics (eg, reimburse health care providers who counsel patients on lifestyle changes) to achieve each strategy.

Three years later, in 2015, *ActiveWV 2015 *was reviewed and revised on the basis of lessons learned from successes and challenges. West Virginia’s experience with this program can inform other states considering creating a stand-alone PA plan. The community of focus was defined by geography (entire state of West Virginia) and topic of interest (PA). Specific objectives were to engage state organizational stakeholders and community implementation stakeholders in designing and implementing the WVPAP. In writing this article, we had 2 objectives: present the results and recommendations from the evaluability assessment (EA) of the WVPAP and describe the lessons learned during the stakeholder activities to revise the WVPAP.

## Methods

### Evaluability assessment

The challenges in evaluating the NPAP described by Kohl et al (6) suggest that a “rush to evaluate” is common in large, multicomponent, multidisciplinary public health programs. To reduce this rush to evaluate, EA was developed in the 1970s to address the challenges that arise from evaluating programs prematurely (16,17). EA is used in public health as a pre-evaluation to determine whether a program is ready for full evaluation, assist program planners in identifying needed program improvements, and ensure that an evaluation will produce useful information (16,17). Multiple models are used for EA, including a 6-step model by Wholey (18) and a 10-step model by Smith (19). Regardless of which model is used, an EA is a cyclical and iterative process with common elements, which include reviewing the following: program documentation (ie, evidence of the program’s validity), program-generated documents, and any guiding logic model or theory of change. In addition, stakeholders are highly engaged, and program staff is interviewed so EA staff can understand the day-to-day reality of running the program (17). Typical end products of an EA include an assessment of 1) the plausibility of achieving desired program outcomes, 2) areas of the program that need further development, 3) feasibility of conducting a full evaluation, 4) options or suggestions for further evaluation, and 5) a critique of the quality and availability of program data (17,19).

### Community engagement

An evaluation team of 4 public health graduate students (including S.S. and S.O.) supervised by a faculty member (C.G.A.) conducted an EA of the WVPAP using a mixed methods approach. Of the 10 EA steps in Smith’s model (19), 8 were used; omitted were Smith’s steps 4 and 10. The other 8 steps were categorized into 3 EA stages: 1) organize, 2) engage stakeholders, and 3) assess implementation and recommend next steps (Table 1). The study was approved by West Virginia University Institutional Review Board for the protection of human subjects.

**Table 1:** Activities Conducted as Part of an Evaluability Assessment (EA) of the West Virginia Physical Activity Plan (WVPAP), 2015

Stage	Step (19)	Activities
Organize	Step 1: Determine purpose, secure commitment, and select work group members.	Ensure agreement on EA activities with WVPAP director.
	Step 2: Define boundaries of program to be studied.	Create syllabus; schedule meetings, activities, and deliverables.
	Step 3: Identify and analyze program documents.	Document review.
Omitted from this EA	Step 4: Develop and clarify program theory.	Not applicable.
Engage stakeholders	Step 5: Identify and interview stakeholders.	Conduct telephone interviews with national stakeholders.
	Step 6: Describe stakeholder perceptions of program.	Meet in person with WVPAP Coordinating Committee.
	Step 7: Identify stakeholder needs, concerns, and differences in perceptions.	Meet in person with implementation partner.
		Facilitate in-person discussions between state and local stakeholders at WVPAP Symposium.
Assess implementation and make recommendations	Step 8: Determine plausibility of program model.	Conduct an online survey.
	Step 9: Draw conclusions and recommend actions.	Facilitate in-person discussions between sector team and state and local stakeholders at WVPAP Symposium.
Omitted from this EA	Step 10: Plan specific steps for use of EA data.	Not applicable.

### EA Steps 1–3: Organize

The EA was conducted from January to May of 2015 as part of a graduate course on program evaluation. Before the semester began, the chair of the WVPAP Coordinating Committee (CC) agreed to serve as the client (without a contract or exchange of money) for whom the EA team would work (EA Step 1). Also before the semester, the activities of the EA team, meeting schedules, and deliverables were agreed on (EA Step 2) and incorporated into the course syllabus. One of the initial tasks completed by the EA team was a document review of all relevant WVPAP and NPAP materials in the peer-reviewed and gray literature (EA Step 3).

### EA Steps 5–7: Engage stakeholders

Findings from the document review and feedback from the WVPAP CC were used to select national, state, and local stakeholders. Structured telephone interviews with 4 national stakeholders, 4 in-person meetings with 3 members of the WVPAP CC, and 3 meetings with 2 representatives of a potential implementation partner and an evaluation consultant were conducted by the 4 students on the EA team under the supervision of C.G.A. (EA Step 5). The students also conducted the telephone interviews (following a script), took detailed notes, and compared notes across interviews to determine response themes. Each in-person meeting followed an agenda developed by the EA team, with each meeting building on data gathered during prior meetings. Meeting activities included the following: a presentation to the EA team about the WVPAP by the chair of the CC, an interactive question-and-answer session between the EA team and the WVPAP CC chair, and a discussion about potential uses of the WVPAP by a potential implementation partner. Interviews and discussions yielded information on stakeholders’ perceptions about gaps in knowledge that this EA could fill regarding implementing and evaluating PA plans in general, and the WVPAP specifically (EA Steps 6 and 7).

### EA Steps 8 and 9: Assess implementation and recommend actions

Information gathered through these preliminary activities was used to inform project activities, described in detail below, including 1) assessing the *ActiveWV 2015* implementation activities (EA Step 8); 2) updating the WVPAP using data gathered from stakeholders on the plausibility of WVPAP’s activities achieving desired outcomes (EA Step 8); and 3) recommending implementation and evaluation actions for the revised WVPAP (EA Step 9).

In March 2015 — 3 years after the release of *ActiveWV 2015* — the EA team developed an online survey in consultation with the WVPAP CC. This survey was patterned after the NPAP survey and the process described by Evenson and Satinsky (20). The purpose was to collect qualitative information about policies, programs, and initiatives related to PA throughout the state and quantify such activities in each priority area, sector, strategy, and tactic of the WVPAP. During the week leading up to the second WVPAP Symposium on March 30 and 31, 2015, the EA team invited, by email, 531 local and state stakeholders that were either 2010 WVPAP Symposium attendees or 2015 WVPAP Symposium registrants to complete the online survey. Respondents were asked to describe local or state policies, programs, or initiatives related to PA. Later, respondents were asked to choose the sector, priority area, and tactic(s) within which the policy, program, or initiative could be classified. 

Response to the online survey was low (78/531 = 14.7%). The education and public health sectors produced the most responses (39% and 22%, respectively), accurately representing the high number of attendees from those sectors relative to other sectors at the 2010 and 2015 symposia. Rather than being used as a true representation of activities under way in West Virginia, the data in Table 2 were presented to the sector teams at the 2015 WVPAP Symposium to engage stakeholders in the discussion about revising the WVPAP. Open-ended items in the survey allowed respondents to describe the PA programs that they were aware of or involved in. Respondents described local programs and key state policy changes and collaborations in support of the WVPAP. Despite prompting, respondents were reluctant or unable to report additional metrics requested to evaluate programs under the WVPAP, such as grant dollars, fund allocations, and intervention outcomes. The survey responses were used to identify areas of high and low implementation frequency based on number of activities associated with each strategy by sector (Table 2). This summary of responses was presented to sector team members at the 2015 WVPAP Symposium to solicit feedback about WVPAP implementation.

**Table 2:** Number of Physical Activity Activities in Each Sector by Priority Area: Evaluability Assessment of the West Virginia Physical Activity Plan, 2015

Sector	1: School Programs and Initiatives	2: Public Awareness and Social Marketing	3: Community Engagement and Environment	4: Institutional and Organizational Support	5: Policy
	No. of Activities				
Business and industry	0	0	3	0	0
Education	34	2	12	0	8
Health care	0	2	0	0	0
Mass media	0	0	0	0	5
Nonprofit or volunteer	4	0	1	1	1
Parks, recreation, fitness and sports	2	0	9	1	0
Public health	7	3	10	9	1
Transportation, land use, and community design	0	0	8	0	4

One aspect that distinguishes EA from other planning tools is that an EA assesses the plausibility that a program will meet its objectives based on the design, inputs, and activities outlined in the program’s logic model or theory (17). The WVPAP EA team solicited input from state and local stakeholders during the WVPAP update process in 2015 to evaluate the appropriateness of priority areas, strategies, and measurable outcomes of the WVPAP. Sector teams at the 2015 WVPAP Symposium provided direct input on the plausibility of the WVPAP by assessing whether proposed strategies and tactics would achieve desired outcomes. Specifically, 8 sector teams of 2 to 10 members were asked during a 4-hour work session to update the strategies and tactics for each priority area using a fast-paced self-managing work team approach. This teamwork approach allows intellectual space for insight and advice from all members and is generally used to complete specific tasks (21). Each sector team was presented with the survey results and asked to analyze and interpret the data against the proposed measurable outcomes, strategies, and tactics from *ActiveWV 2015,* one priority area at a time. Because of time limitations at the symposium, the WVPAP CC chose to enforce strict time limits, which is not a traditional approach to conducting self-managing work teams. Each sector team was given the task of 1) deciding whether to keep, revise, or remove the strategies and tactics for each priority area and 2) identifying which measurable outcome(s) each strategy would work toward in *ActiveWV 2020*. Each sector team was facilitated by a member of the WVPAP CC or EA team. Results were immediately reported orally to all attendees, and notes were taken for each sector team by a member of the WVPAP CC or EA team. These notes were reviewed by the WVPAP CC after the symposium when updating the WVPAP.

The survey and plan update activities helped the EA team form recommendations for sector teams and the WVPAP CC to use in implementing and evaluating the revised plan. These recommendations were developed by the EA team independent of the WVPAP CC and presented to the WVPAP CC, a private evaluation consultant, and the West Virginia Community Development Hub (The Hub) — a nonprofit and potential implementation partner that was developing a network of local PA advocates throughout West Virginia concurrent with the WVPAP revision process.

## Outcome

Using the results of stakeholder engagement activities as a basis, the EA team recommended 5 action steps for the WVPAP CC to take when implementing, revising, or evaluating future plans. Other states’ plans to increase PA could also benefit from these recommendations, which are 1) establish an implementation entity to work specifically on local plans, 2) create sector-specific logic models to help local planning, 3) evaluate implementation frequently from the outset, 4) recognize that planning can be quick and efficient; and 5) understand what constitutes a good plan to increase population PA. Each recommendation is described in more detail below.

**First recommendation: Appoint an entity to develop an implementation plan.** Two representatives from The Hub were among our key stakeholders; they reported being overwhelmed by the WVPAP, similar to findings reported elsewhere about the NPAP (5). The Hub specializes in community development, including creating local food systems and engaging community members in the development process. The representatives recommended that implementation focus on a narrow geographic area by developing county or city PA plans and engaging local community members in using the new WVPAP as a source of activities from which to pick and accomplish during a specific period. This approach is similar to evaluation recommendations about state and local use of the NPAP in Texas (20,22).

**Second recommendation: Create logic models for each sector to facilitate planning and simplify the WVPAP for community stakeholders.** The implementation entity should create these logic models in collaboration with the local program’s stakeholders and evaluation team. A good approach to this task is to work from right to left on each logic model, beginning with the measurable outcome (on the right side of logic model) and working from there to the left to select trackable outputs, specific activities, and responsible parties and inputs needed to meet overarching measurable outcomes in each priority area. For example, “Increase the funds used to improve pedestrian and bicycle infrastructure” was an outcome for measuring success in priority area 3, community engagement and environment. To assess success, evaluators should work with implementation staff and the transportation sector to select the outputs (eg, miles of bicycle lanes created), activities (eg, maps of bicycle lanes produced), and inputs (eg, create bicycle advisory group) necessary to achieve the desired outcome.

**Third recommendation: Have an independent evaluator prospectively evaluate *ActiveWV 2020* implementation activities.** Our attempt to assess implementation activities via the online survey highlighted how difficult it is to generate responses retrospectively from a diverse set of unfunded partners. In concert with the development of logic models, potential evaluation data sources and activities should be developed. These data collection methods include 1) a performance monitoring tool to track implementation activities similar to the way NPAP was tracked during its first year (20) and 2) a system for extracting data from policies or plans in order to compare them with best practices. One person should be selected to collect and enter the information on each sector — probably the sector team leader or a subleader. Collection should commence from the outset and be repeated frequently to assess the proximal activities and outputs achieved toward meeting the more distal measurable outcome goals. As these data are collected, the WVPAP CC, implementation entity, and evaluator should review data in relation to baseline measurements to determine progress made. These data should be augmented by qualitative data gathered via frequent structured interviews to identify and disseminate successes and ways to address barriers experienced in implementation. Best practices can be explained to sector teams and stakeholders to further inform their activities in implementation. With regard to the WVPAP, the more distal measurable outcomes at the far right of a logic model were already identified by the WVPAP CC with an existing data source in mind so that secondary data analysis or simple primary collection and analysis of online documents (eg, policies, plans) may be used for evaluation.

**Fourth recommendation: Recognize that planning with stakeholders can be quick and efficient.** The fast pace of the self-managing work teams used with sector teams was successful during one afternoon in developing better defined and aligned strategies, tactics, and measurable outcomes. The combination of structure and pace allowed the update to occur with a highly qualified, time-pressed group of stakeholders in one collaborative setting. Adapting this approach as a time-limited activity pushed sector teams to quickly complete a defined task while still allowing for creativity. We recommend this approach, either at the inception or at a revision of a PA plan because stakeholders were productive despite having little time to be away from their primary duties. A caveat to this approach is that it takes extensive planning and work by the team leading the activities to design and implement the process, revise the plan, and disseminate the results.

**Fifth recommendation: Learn what constitutes a good plan to increase population PA.** Developing an evaluation plan for the WVPAP, as the EA team had originally intended, proved difficult because they were attempting to “plan an evaluation of a plan.” The team had to adjust because the WVPAP was a strategic vision rather than an implementation plan. An implementation plan, with specific activities and responsible parties identified, is more “evaluation ready” and easily translated into an evaluation plan than is a strategic vision. The EA process proved valuable because it allowed the EA team to develop recommendations for implementing and evaluating future activities of the WVPAP rather than to create an evaluation plan prematurely. Other state and local PA programs should not take this lesson lightly because trying to create an evaluation plan prematurely also created implementation challenges. Feedback from implementation partners and sector teams suggested that they wanted the WVPAP to include a more specific implementation plan — or a menu of suggestions — from which to pick an activity to perform.

## Interpretation

Our state community engagement project, the EA described in this article, highlights the need to simplify the state PAP to make it a more locally driven process that engages communities in implementation. Specifically, because of the comprehensive, visionary nature of the WVPAP, attempts to simplify the plan into a single logic model and identify specific implementation activities were unsuccessful. There are 2 key lessons from this. First, rather than develop a logic model that encompasses all sectors and strategies of the entire WVPAP, the EA team’s recommendation is to simplify the process by creating 1 or more logic models for each sector as a way to unify each sector on specific implementation activities and outcomes. Second, these sector-specific logic models could be used to further simplify the WVPAP so it could be used as a menu of suggested activities from which a community could choose the activities best suited for it. The recommendations and lessons learned from the EA process described herein may serve to guide other national, state, or local programs to develop or revise their PA plan. The EA process proved flexible and valuable in engaging community stakeholders in developing implementation and evaluation recommendations for the next iteration of the WVPAP, and we recommend EA as a way to advance planning for PA programs.
